# Effects of Stromal Cell-Derived Factor-1*α* Secreted in Degenerative Intervertebral Disc on Activation and Recruitment of Nucleus Pulposus-Derived Stem Cells

**DOI:** 10.1155/2019/9147835

**Published:** 2019-11-19

**Authors:** Jinwei Ying, Zhihua Han, Shishen Pei, Linghao Su, Dike Ruan

**Affiliations:** ^1^Department of Orthopedic Surgery, The First Affiliated Hospital of Wenzhou Medical University, Wenzhou 325000, China; ^2^Department of Orthopedic Surgery, The Sixth Medical Center of PLA General Hospital, Beijing 100048, China; ^3^The Second School of Clinical Medicine, Southern Medical University, Guangzhou 510515, China; ^4^Experimental Trauma & Orthopedic Surgery, JW Goethe-University, Frankfurt am Main, Germany; ^5^Department of Trauma and Orthopedics, Trauma Center, Shanghai General Hospital, School of Medicine, Shanghai Jiao Tong University, Shanghai 201620, China; ^6^Department of Orthopedic Surgery, The Fourth People's Hospital of Hengshui, Hengshui 053000, China

## Abstract

Stromal cell-derived factor-1*α* (SDF-1*α*) plays a significant role in mobilizing and recruiting mesenchymal stem cells (MSCs) to the sites of injury. This study investigated the potential of SDF-1*α* released in the degenerative intervertebral disc (IVD) to activate and recruit endogenous nucleus pulposus-derived stem cells (NPSCs) for regeneration *in situ*. We found SDF-1*α* was highly expressed and secreted by the native disc cells when cultured in the proinflammatory mediators *in vitro* mimicking the degenerative settings. Immunohistochemical staining also showed that the expression level of SDF-1*α* was significantly higher in the degenerative group compared to that in the normal group. In addition to enhancement of viability, SDF-1*α* significantly increased the number of NPSCs migrating into the center of the nucleotomized bovine IVD *ex vivo*. After the systemic delivery of exogenous PKH26-labelled NPSCs into the rats *in vivo*, there was a significant difference in the distribution of the migrated cells between the normal and the degenerative IVDs, which might be caused by the different expression levels of SDF-1*α*. However, blocking CXC chemokine receptor 4 (CXCR4) with AMD3100 effectively abrogated SDF-1*α*-stimulated proliferation and migration. Taken together, SDF-1*α* may be a key chemoattractant that is highly produced in response to the degenerative changes, which can be used to enhance the proliferation and recruitment of endogenous stem cells into the IVDs. These findings may be of importance for understanding IVD regenerative mechanisms and development of regenerative strategies *in situ* for IVD degeneration.

## 1. Introduction

Intervertebral disc (IVD) degeneration is one major cause of low back pain (LBP) in the modern society [1, 2]. It is characterized by chronically increased levels of numerous proinflammatory factors secreted by the native disc cells that promote matrix degradation, chemokine production, and cell phenotype changes [3]. Release of chemokines from the degenerative IVD promotes the activation and infiltration of immune cells, amplifying the inflammatory cascade [3]. However, some of these chemokines have also been shown to be involved in the IVD self-repairing process by activation and recruitment of endogenous disc cells [4]. It had been generally accepted that cartilaginous tissues have a limited self-repairing capacity in adult mammals [5, 6]. However, recent evidence suggests that the endogenous stem cells residing in the IVD contribute to early regeneration of IVD [7]. Many researchers have also demonstrated the presence of nucleus pulposus- (NP-) derived stem cells (NPSCs) among various species, possessing the capacity of chondrogenic differentiation similar to other mesenchymal stem cells (MSCs) [8, 9]. Previously, our research group also successfully isolated and identified the endogenous NPSCs from human lumbar IVDs [10] and rat coccygeal IVDs [11]. In this context, it is meaningful to investigate the role of chemokines in recruiting NPSCs into the pathological sites for self-repairing the degenerative IVD.

A number of studies have shown that the chemokine stromal cell-derived factor-1*α* (SDF-1*α*, also known as CXCL12) is upregulated in the injured sites and plays an important role in mobilization and recruitment of circulating or residing MSCs through interaction with its cognate receptor CXC chemokine receptor 4 (CXCR4) [12, 13]. SDF-1*α* was firstly reported to be continuously secreted by bone marrow stromal cells, which has the strong chemotaxis to stem cells with the receptor CXCR4 [14, 15]. Therefore, the SDF-1/CXCR4 axis is responsible for the homing of MSCs or hematopoietic stem cell (HSCs) to the bone marrow [16]. Mobilization is the opposite direction migration relative to homing. The mechanism of AMD3100 on the mobilization of MSCs or HSCs has been basically clarified. Some researchers confirmed that AMD3100 as a specific antagonist of SDF-1*α* ligand blocks the SDF-1/CXCR4 interaction and the downstream signaling and then synergistically downregulates the expression of adhesion molecules [17]. As the result, the highly expressed SDF-1*α* in the bone marrow microenvironment loses the chemotaxis to MSCs or HSCs. Theoretically, AMD3100 can be an effective mobilizer for MSC or HSC migration into the peripheral blood circulation.

It was documented that the increased SDF-1*α* in the osteoarthritis tissue could promote the recruitment of CXCR4-positive MSCs into the injured cartilage [18]. The expression of SDF-1*α* was also reported to be upregulated in the human degenerative IVD [19, 20], and overexpression of its receptor CXCR4 could promote MSC retention in the degenerative IVD and enhance stem cell-based IVD regeneration [21]. In addition, the hyaluronan-based delivery of SDF-1*α* significantly boosted the recruitment of MSCs into the degenerative IVD in an *ex vivo* organ culture [22]. However, stem cells recruited into IVD appear more challenging because the circulating MSCs need to migrate over longer distances to reach the inner structure of IVD due to its avascular nature.

Based on these findings, we hypothesize that the SDF-1/CXCR4 axis might play a crucial role in the activation and recruitment of the endogenous NPSCs contributing to IVD regeneration *in situ*. In the present study, we measured the expression level of SDF-1*α* in the degenerative condition and evaluated the potential of SDF-1*α* as a chemoattractant to recruit NPSCs into an *ex vivo* degenerative IVD organ model. In addition, systemic delivery of exogenous NPSCs into the rats was performed to understand the effect of expression distribution of SDF-1*α* in the degenerative IVD on the transplanted NPSCs *in vivo*.

## 2. Materials and Methods

### 2.1. Ethics Statements

All experiments and surgical procedures were performed in accordance with Southern Medical University guidelines and approved by the Laboratory Animal Ethics Committee of the Sixth Medical Center of PLA General Hospital, Beijing, China (IEC2018046).

### 2.2. Isolation and Culture of NPSCs and Nucleus Pulposus Cells (NPCs)

The NP tissue from the coccygeal IVDs of 10 adult male Sprague-Dawley (SD) rats (250-300 g) was collected and cut into two halves for isolation of NPSCs and NPCs, respectively. NPSCs were isolated by a differential adhesion method as previously described [23]. Briefly, the nonadherent cells floating in the medium were removed after 24 h of culture and the adherent cells were expanded in Dulbecco's modified Eagle medium/F12 (DMEM/F12; Hyclone, Logan, Utah, USA) supplemented with 10% fetal bovine serum (FBS; Gibco, Grand Island, NY, USA) and 1% Pen/Strep (Solarbio, Beijing, China), while the culture medium was changed every 3 days. However, for the isolation of NPCs, the medium was removed with suspension cells and fragments after 3 days, and the adherent cells were fed by complete replacement of standard medium every 3 days. All cells were passaged when they reach 80-90% confluence using 0.05% trypsin-EDTA (Gibco) at a ratio of 1 : 3. Cells at passage 3 (P3) were used for subsequent experiments.

### 2.3. Effect of SDF-1*α* on Cell Viability of NPSCs

#### 2.3.1. Cell Counting Kit-8 (CCK-8) Assay

We seeded NPSCs into 96-well plates (Costar, Cambridge, MA, USA) at a density of 2 × 10^3^ cells/well and then applied 0, 25, 50, and 100 ng/mL SDF-1*α* (PeproTech, Rocky Hill, NJ, USA) in 100 *μ*L complete medium. For inhibition experiment, we pretreated cells with the inhibitor AMD3100 (10 *μ*g/mL, Sigma-Aldrich, St. Louis, MO, USA) for 2 h before exposing them to 100 ng/mL SDF-1*α*. The cell proliferation was evaluated at 1, 3, 5, and 7 days of culture using CCK-8 assay (Dojindo, Kumamoto, Japan). The absorbance at 450 nm was measured with a microplate reader (Bio-Rad, California, USA). The mean growth was created by three independent experiments at different time points.

#### 2.3.2. 5-Ethynyl-2′-deoxyuridine (EdU) Incorporation Assay

The quantification of cell proliferation was performed through EdU incorporation assay using BeyoClick™ EdU-594 Cell Proliferation Kit (Beyotime, Shanghai, China) according to the manufacturer's instruction. Particularly, cells were treated with 10 *μ*M EdU and incubated for 2 h at 37°C. Then, the cells were fixed with 4% formaldehyde and permeabilized with 0.3% Triton X-100. Fixed cells were washed with PBS three times and incubated with Click Reaction Solution containing Azide 594 for 30 min in the dark before counterstaining nuclei with Hoechst 33342 (Beyotime). The EdU-positive rate of each group was assessed by selecting three random fields under a fluorescent microscope (CFM-300, Nikon, Japan) and measured using Image-Pro Plus 6.0 software (Media Cybernetics, Silver Spring, MD, USA); then the mean values were used for analysis.

### 2.4. Activation of NPCs to Synthesize and Secrete SDF-1*α* in the Proinflammatory Culture In Vitro

To mimic the proinflammatory microenvironment of the degenerative IVD, NPCs (1 × 10^5^ cells/well) were incubated in serum-free medium containing 10 ng/mL IL-1*β* (PeproTech, Rocky Hill, NJ, USA) and 50 ng/mL TNF-*α* (PeproTech) for 48 h. The secreted SDF-1*α* in the supernatant was evaluated using enzyme-linked immunosorbent assay (ELISA), while the adherent NPCs were used for real-time RT-PCR.

### 2.5. SDF-1*α*-Stimulated Migration Capacity of NPSCs Ex Vivo

#### 2.5.1. IVD Organ Culture

**Figure 1:**
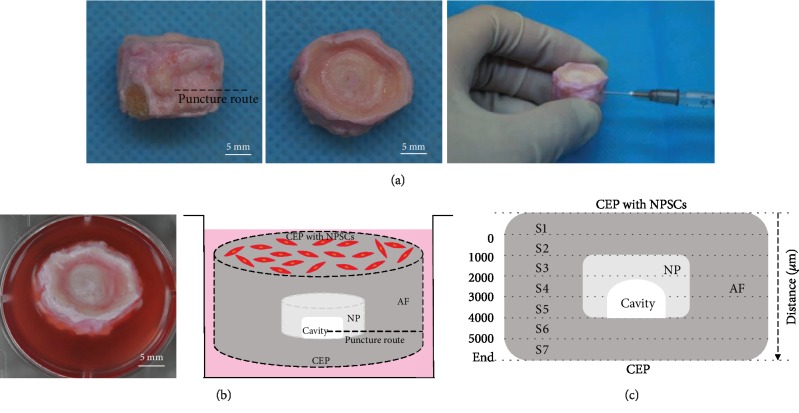
(a) Representative images of the process of establishing an *ex vivo* organ culture model using the bovine caudal IVD. For cell migration assay *ex vivo*, IVD was punctured in the coronal plane and aspirated out some NP tissue (0.08-0.15 cm^3^) close to the lower CEP with a 22-gauge needle to create a cavity and then positioned into the culture plate with the upper CEP side up. (b) Schematic representation of the experimental design of cell migration assay *ex vivo*. PKH26-labelled NPSCs were cultured on the top of the upper CEP and allowed to migrate for 48 h. (c) Schematic view of the regional division of the IVD for cell migration assessment. The sagittal plane of IVD was sectioned into 1 mm height subsections from top to bottom in order to evaluate the depth of cell migration. IVD: intervertebral disc; NP: nucleus pulposus; CEP cartilaginous endplate; AF: annular fibrosus; NPSCs: nucleus pulposus-derived stem cells.

In order to evaluate the recruitment effect of SDF-1*α* on NPSCs *ex vivo*, an organ culture study was performed following the protocol as previously described [24]. Three bovine tails (5-8 months old) were harvested from a local abattoir within 3 h of death. Briefly, five caudal IVD units with cartilaginous endplates (CEPs) were excised from each bovine tail, then rinsed in PBS containing 10% Pen/Strep, and the osseous debris was further removed from CEPs using a jet lavage system (Zimmer, Winterthur, Switzerland). To induce IVD degeneration, each disc was punched with a 22-gauge needle and the NP tissue was partially aspirated to leave a cavity (0.08-0.15 cm^3^/per tissue, Figure 1(a)). Thereafter, all IVDs was injected with 100 *μ*L SDF-1*α* solution in PBS at different concentrations (0, 25, 50, and 100 ng/mL), then incubated in IVD culture medium overnight at 37°C, 85% humidity, and 5% CO_2_.

To inhibit SDF-1*α* binding to CXCR4, NPSCs were preincubated with 10 *μ*g/mL AMD3100 for 2 h. The IVD culture medium was composed of DMEM/F12 supplemented with 2% FBS, 1% Pen/Strep, 1% insulin-transferrin-selenium (ITS; BD, USA), and 0.1% Primocin (Invivogen, Hong Kong). Five motion units from each tail were randomly assigned into the five groups (three IVDs per group).

#### 2.5.2. Cell Recruitment Assay

We labelled NPSCs with PKH26 (red) (PKH Fluorescent Cell Linker kit, Sigma-Aldrich) following the manufacturer's instruction, then applied 30 *μ*L PKH26-NPSC suspension (1 × 10^6^ cells) on the top of the upper CEPs and incubated the IVDs for 30 min to allow cells to adhere. Afterward, IVD culture medium was added to reach the surface of the upper CEPs (Figure 1(b)). The study by Pereira et al. [22] showed that the highest concentration of SDF-1*α* released in the *ex vivo* culture medium was obtained at 48 h, although the amount detected was lower than the theoretical maximum amount that could be released. Accordingly, after 48 h incubation for cell migration, IVDs were rinsed in PBS and incubated with trypsin to remove the cells that attached to the surface and then fixed in 4% buffered formalin.

#### 2.5.3. Cell Recruitment Analysis

After decalcification, the IVDs were cut in a sagittal plane with a thickness of 5 *μ*m. Cell recruitment was revealed by DAPI staining (Beyotime, Shanghai, China). The images from three random midsagittal sections of each specimen were acquired under a motorized microscope (Axio Scan Z1; Zeiss, Jena, Germany) at 5x magnification. Then cell counting was manually performed using ZEN 2012 software (Blue Edition, Zeiss) following the criteria below:

1. The coverage of the IVD through the whole sagittal section included CEPs, AF, and NP
2. The cells retained on the upper CEPs that seeded initially were excluded
3. The IVD sections were divided into 1 mm height subsections (S1, S2, S3, S4, S5, S6, and S7) (Figure 1(c))
4. The total number of migrated cells in the whole sagittal IVD section was defined as the mean number of migrated cells in the three random midsagittal sections
5. The average percentage of cell migration in one subsection was obtained by the number of cells migrated into the subsection divided by the total number of cells migrated in the whole sagittal section (×100%)

### 2.6. SDF-1*α*-Stimulated Migration Capacity of NPSCs In Vivo

#### 2.6.1. Animal Model of IVD Degeneration

Fifteen male SD rats (2-3 months) were randomly divided into three groups (*n* = 5). Group A without treatment was regarded as the control group. Two consecutive coccygeal IVDs (Co7/Co8 and Co8/Co9) of animals in group B and group C were induced to degeneration using a needle puncture method described previously [25].

#### 2.6.2. In Vivo Chemotaxis Assay

**Figure 2:**
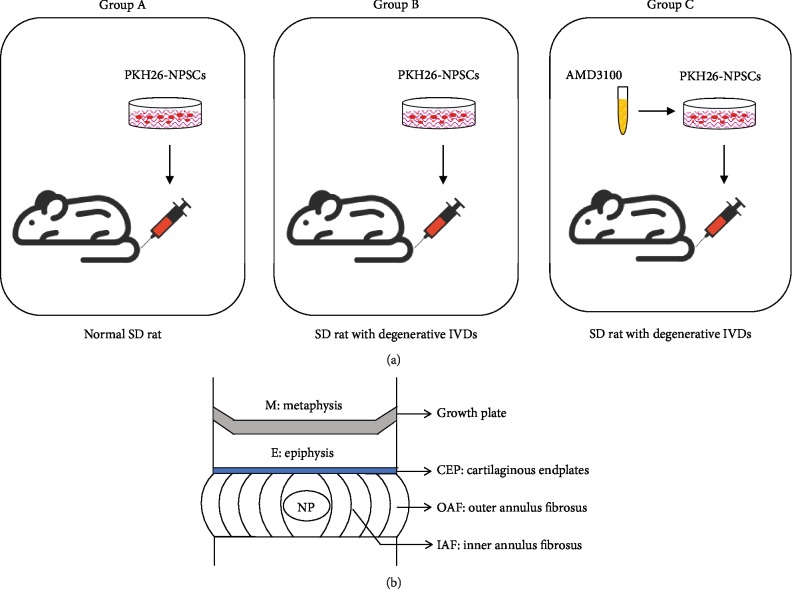
(a) Illustrative description of the three different groups for *in vivo* migration of exogenous NPSCs. (b) Schematic diagram of the distribution areas of the migrated cells in the rat coccygeal IVDs. NPSCs: nucleus pulposus-derived stem cells; IVD: intervertebral disc.

Four weeks later, rats in group A and group B were systematically given 1 × 10^6^ PKH26-NPSCs in a 500 *μ*L aliquot of PBS via injection into the tail vein (Figure 2(a)). Meanwhile, PKH26-NPSCs pretreated with 10 *μ*g/mL AMD3100 were given to the animals in group C (Figure 2(a)).

All animals were kept for two weeks after treatment, then euthanized for sample collection. The coccygeal IVDs that excised in continuity with adjacent vertebral bodies were processed for histological evaluation. In short, after fixation, rapid decalcification, and embedding, the samples were then cut through the sagittal plane at 5 *μ*m thickness, counterstained by DAPI, and briefly immersed in ethanol and xylene. Lastly, the sections were detected under a fluorescence microscope (Axio Scan Z1).

To identify NPSC migration distance and distribution from the bone marrow of the adjacent vertebral body, the IVD was divided into six areas according to the method described by Sakai et al. [26]: metaphysis (M), epiphysis (E), CEP, outer annulus fibrosus (OAF), inner annulus fibrosus (IAF), and NP (Figure 2(b)). The cell density of PKH26-labelled NPSCs in each area was calculated from 5 randomly selected serial sections (cells/mm^2^) using Image-Pro Plus 6.0 software (Media Cybernetics, Silver Spring, MD, USA). The appearance rate of migrated NPSCs in one certain area was calculated; the cell density of NPSCs counted in the area (cells/mm^2^) was divided by the density of NPSCs counted in area M (cells/mm^2^) × 100%.

### 2.7. Real-Time RT-PCR

After extracting total RNA, cDNA was synthesized by reverse-transcription with Quantscript RT Kit (TIANGEN Biotech, Beijing, China) following the manufacturer's instructions. Then the obtained cDNA was used for a real-time PCR test using SYBR® Premix Ex TaqTM (Tli RNaseH Plus) (TaKaRa, Otsu, Japan). All samples were amplified in duplicates with a CFX96 Touch Real Time PCR Detection System (BioRad, Puchheim, Germany) using rat gene-specific primers as follows: SDF-1 (NC_005103.4) (forward: 5′-TTCTATTGAGGACTAGCACGTC-3′, reverse: 5′-CTGTCCTAAGGAAAGGTAGGTG-3′); CXCR4 (NC_005112.4) (forward: 5′-GCTCCAGAATGTGTGGTAAATC-3′, reverse: 5′-CACCAAGCAAGTTTACCATTGA-3′); and *β*-actin (NC_005111.4) (forward: 5′-GGAGATTACTGCCCTGGCTCCTA-3′, reverse: 5′-GGAGATTACTGCCCTGGCTCCTA-3′). *β*-Actin was used as the reference gene. The cycle threshold (Ct) values of triplicate samples were averaged for analysis. The relative gene expression was calculated using the 2^–*ΔΔ*Ct^ method and was normalized to the house keeping gene expression.

### 2.8. Elisa

The level of SDF-1*α* in the cell culture supernatant was measured by a multi-detection microplate reader (Bio-Rad, California, USA) using a double-antibody sandwich ELISA kit (USCN, Wuhan, China) according to the manufacturer's protocol.

### 2.9. Immunohistochemistry

The specimens were fixed for one week in 10% formaldehyde, decalcified for four weeks in 0.05% EDTA, and then embedded in paraffin. Serial sagittal sections of IVDs (5 *μ*m thickness) were prepared, deparaffinized in dimethylbenzene, and rehydrated. Some sections were stained with hematoxylin and eosin (H&E) and Alcian Blue to evaluate the severity of degeneration. For immunohistochemical staining, other sections were incubated with anti SDF-1 (1 : 100 dilution; Cell Signaling Technology, MA, USA) overnight at 4°C. On the second day, all sections were washed with PBS and were incubated with the secondary antibody conjugated with horseradish peroxidase (1 : 200 dilution; Abcam, MA, USA) at 37°C for 1 h, followed by color development with diaminobenzidine (DAB, Beyotime). Then the sections were counterstained with hematoxylin (Beyotime). The stained sections were examined using a light microscope (Nikon).

### 2.10. Immunofluorescence

NPSCs seeded on the slides were treated with or without SDF-1*α* (100 ng/mL) and SDF-1/CXCR4 axis inhibitor AMD3100 (10 *μ*g/mL). Then cells were fixed, permeabilized, and blocked with 10% goat serum. Subsequently, they were incubated with the primary antibody CXCR4 (1 : 50 dilution; Abcam) at 4°C overnight. The next day, cells were washed with PBS and then incubated with FITC-conjugated secondary antibody (Abcam) and fluorescence-conjugated phalloidin working solution (CytoPainter F-actin staining kit, Abcam) for 60 min at room temperature. DAPI (Beyotime) was used for counterstaining. Images were obtained with a confocal laser scanning microscope (LSM780, Zeiss).

### 2.11. Western Blotting

Protein samples were separated by 10% sodium dodecyl sulfate-polyacrylamide gel (SDS-PAGE) electrophoresis and electrotransferred onto polyvinylidene fluoride (PVDF) membranes (Millipore, Billerica, MA, USA). After blocking with 5% nonfat milk for 2 h at room temperature, the membranes were incubated with primary antibodies CXCR4 (1 : 100 dilution) and GAPDH (1 : 10000 dilution; Abcam) at 4°C overnight. The membranes were then incubated with horseradish peroxidase-conjugated secondary antibody (Abcam) for 1 h at room temperature. Immunolabeling was visualized using an electrochemiluminescence (ECL) reagent (Millipore). The relative protein intensities were measured using Image-Pro Plus 6.0 software.

### 2.12. Statistical Analysis

The data are expressed as the mean ± standard deviation (SD) and analyzed using SPSS 15.0 software (Chicago, Illinois, USA). The significance of the differences among different groups was determined by one-way analysis of variance (ANOVA) followed by the Student-Newman-Keuls test for post hoc multiple comparisons. *P* < 0.05 was considered statistically significant.

## 3. Results

### 3.1. Effect of SDF-1*α* on the Proliferation of NPSCs

**Figure 3:**
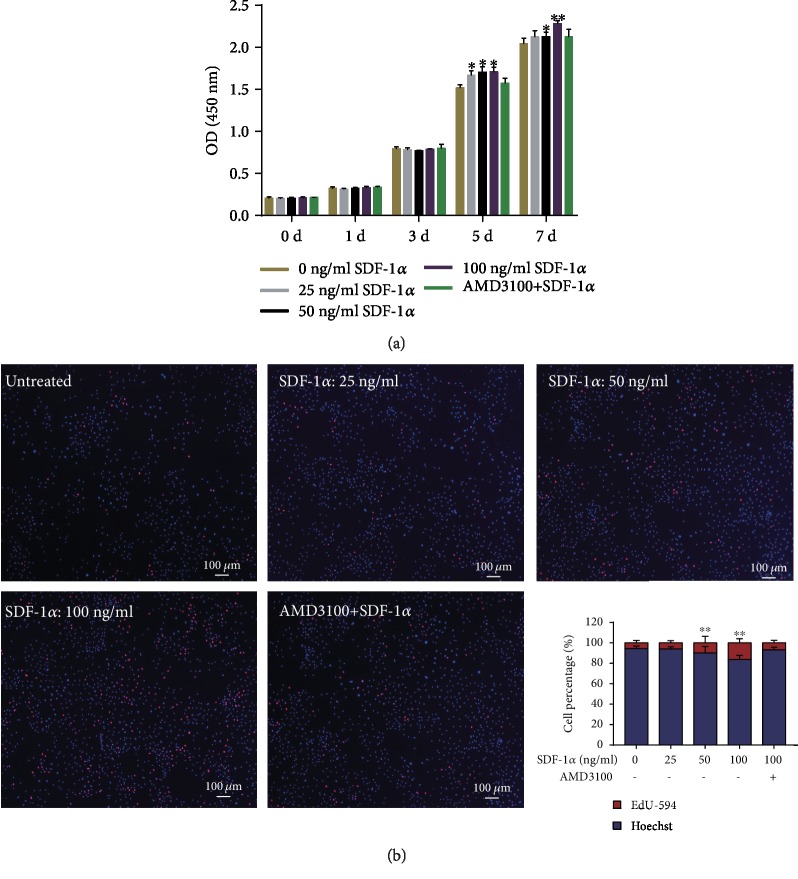
Effect of SDF-1*α* on the proliferation of NPSCs. (a) CCK-8 assay demonstrating the proliferation capacities of NPSCs treated with different concentrations of SDF-1*α* after 1, 3, 5, and 7 days of culture, respectively. (b) EdU incorporation assay showing the proliferation rates of NPSCs treated with different concentrations of SDF-1*α* after 72 h. Red indicates the proliferating cells. SDF-1*α*: stromal cell-derived factor-1*α*; NPSCs: nucleus pulposus-derived stem cells; CCK-8: cell counting kit-8; EdU: 5-ethynyl-2′-deoxyuridine. Data are expressed as the mean ± SD (*n* = 3); ^∗^*P* < 0.05 and ^∗∗^*P* < 0.001.

CCK-8 assay was used to analyze the proliferation of NPSCs in response to SDF-1*α*. The result showed that the proliferation capacity of NPSCs was not affected by SDF-1*α* until the 5th day. On day 5 and day 7, the proliferation of NPSCs was significantly enhanced in a dose-dependent manner (*P* < 0.05) (Figure 3(a)). Similarly, EdU incorporation assay also confirmed that the proliferation ratio of NPSCs gradually raised with the increased SDF-1*α* (Figure 3(b)). However, antagonizing CXCR4 with AMD3100 significantly inhibited the SDF-*α*-induced proliferation of NPSCs.

### 3.2. SDF-1*α* Expression in the Degenerative IVD Microenvironment

**Figure 4:**
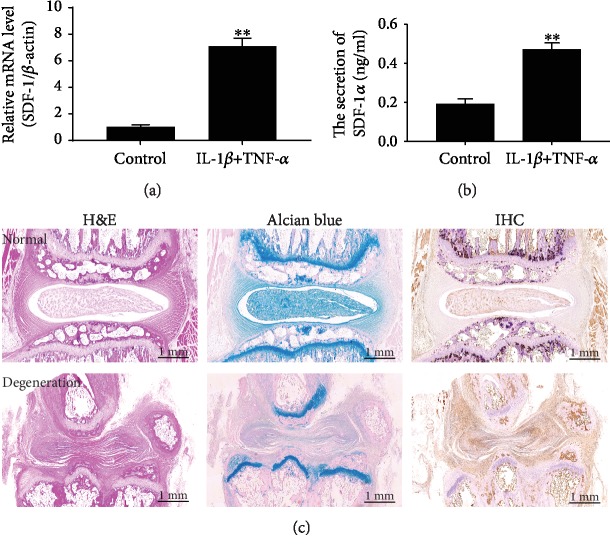
The expression of SDF-1*α* in the degenerative IVD condition. The levels of synthetic mRNA (a) and secreted protein (b) of SDF-1*α* from NPCs after treatment with the proinflammatory cytokines IL-1*β* and TNF-*α in vitro*. (c) Histological analysis of the relationship between IVD degeneration and SDF-1*α* expression. SDF-1*α*: stromal cell-derived factor-1*α*; IVD: intervertebral disc; NPCs: nucleus pulposus cells; IL-1*β*: interleukin-1*β*; TNF-*α*: tumor necrosis factor-*α*; H&E: hematoxylin and eosin; IHC: immunohistochemistry. Data are expressed as the mean ± SD (*n* = 3); ^∗∗^*P* < 0.001.

We performed not only real-time RT-PCR and ELISA to examine the mRNA synthesis and protein secretion of the chemokine SDF-1*α* from NPCs after treatment with proinflammatory cytokines but also immunohistochemical analysis to compare the expression of SDF-1*α* between the normal and degenerative groups. The results showed that both synthetic mRNA and secreted protein of SDF-1*α* from NPCs after treatments with IL-1*β* and TNF-*α* were significantly increased *in vitro* (*P* < 0.001) (Figures 4(a) and 4(b)). In addition, histological analysis showed that the IVD degeneration model was induced successfully due to the loss of IVD height and proteoglycan and the disorganized structure. However, the expression of SDF-1*α* in the degenerative IVD was significantly higher than that in the control group (Figure 4(c)).

### 3.3. Effect of SDF-1*α* on NPSC Migration in an IVD Organ Culture Model Ex Vivo

**Figure 5:**
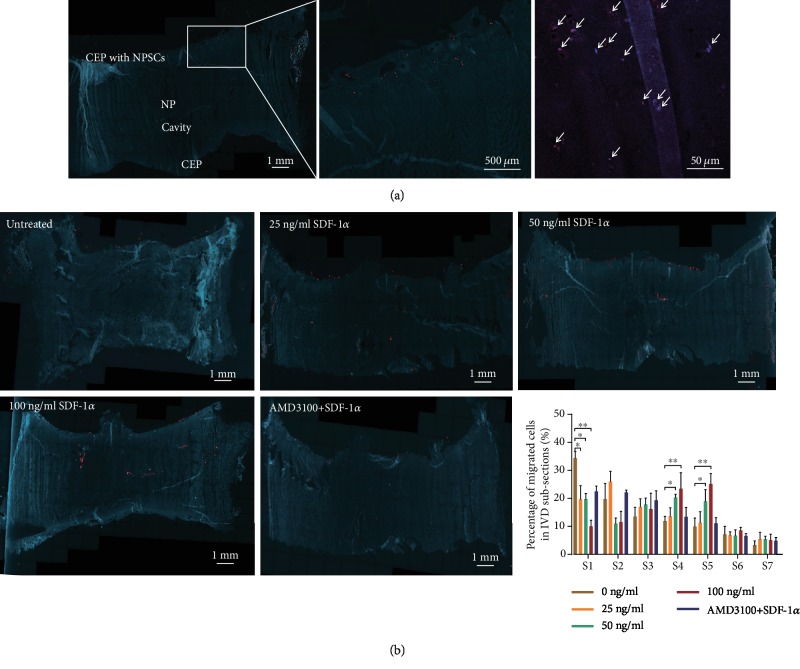
NPSC migration toward SDF-1*α* in an *ex vivo* IVD organ culture model. (a) Identification of the NPSCs migrated into the IVD organ culture model *ex vivo*. On the sagittal section, PKH26-labelled cells in red could be observed from the upper CEP to the cavity. Arrows indicate the migrated NPSCs into the bovine caudal IVD. (b) Comparison of the distribution of the migrated cells among the different groups. NPSCs: nucleus pulposus-derived stem cells; SDF-1*α*: stromal cell-derived factor-1*α*; IVD: intervertebral disc; CEP: cartilaginous endplate; NP: nucleus pulposus. Data are presented as the mean ± SD (*n* = 3); ^∗^*P* < 0.05 and ^∗∗^*P* < 0.001.

NPSC migration ability *ex vivo* was analyzed by observing the presence and distribution of PKH26- (red) labelled cells in the bovine caudal IVDs under the fluorescence microscope (Figure 5(a)). To eliminate the possibility of the cells on the upper CEPs to be dragged into the center of IVDs by the blade during the cutting procedure, the adherent cells on the upper CEPs were digested off by trypsin before. Scarcely, any cells were found on the upper surface, which confirmed that NPSCs had been removed thoroughly and it was effectively able to evaluate NPSC migration using this organ culture model *ex vivo*. Concerning the migration profile of NPSCs in the IVDs, we discriminated the proportion of the migrated cells in the subsections at different depths (Figure 1(c)). As shown in Figure 5(b), in the control group without any treatment, we observed that the number of migrated cells gradually decreased with the increased depth, a significantly higher number of cells in the first subsection (S1) compared to other experimental groups (*P* < 0.05). No statistical differences among the groups were observed in the following subsections (S2 and S3) (*P* > 0.05), although 25 ng/mL SDF-1*α* could attract more NPSCs into S2 section than other groups. In the middle subsections (S4 and S5), the effect of SDF-1*α* on the enhancement of cell recruitment was more evident, with higher numbers of NPSCs being detected in these subsections in the groups treated with 50 ng/mL and 100 ng/mL SDF-1*α* (*P* < 0.05). Similarly, the number of migrated cells in the last subsection (S7) was the least in all the groups. It suggests that NPSCs have the capacity to migrate through CEPs and extracellular matrix, and cell recruitment is enhanced by SDF-1*α* in the IVD tissue.

### 3.4. Effect of SDF-1*α* on NPSC Migration in the Degenerative IVD Model In Vivo

**Figure 6:**
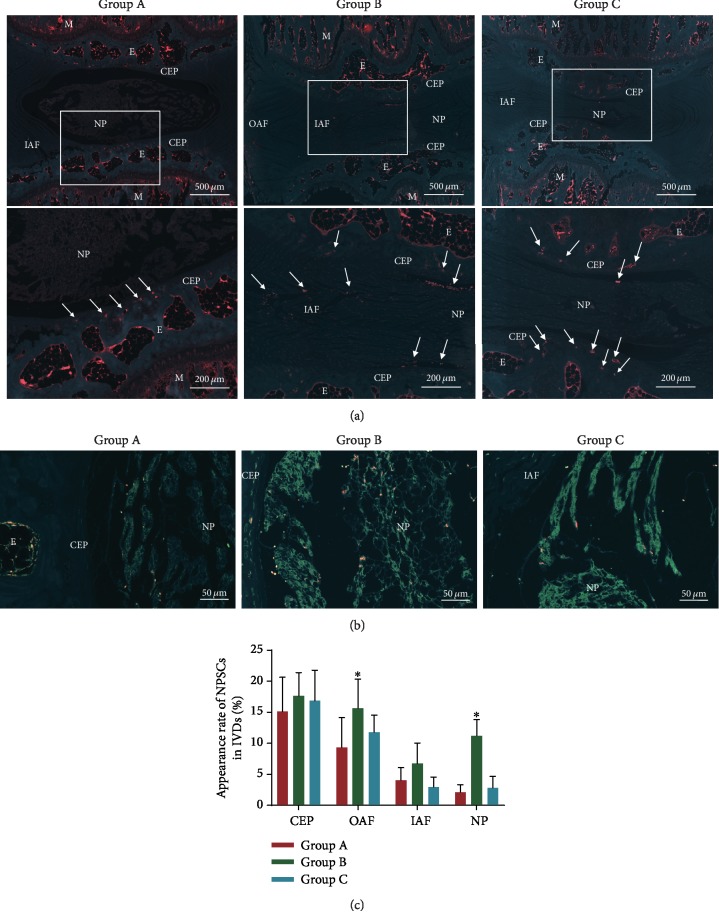
Exogenous NPSC migration into the rat coccygeal IVDs *in vivo* after systemic delivery. (a) The migration of PKH26-labelled NPSCs into the IVDs at different groups under the fluorescence microscope. Arrow indicates the PHK-26-labelled NPSCs migrated into the IVD tissue. (b) The representative immunofluorescence demonstrating the correlation between the migration profile of exogenous NPSCs and the distribution of SDF-1*α* highly secreted in the degenerative IVD. Green fluorescence represents SDF-1*α* expressed in the IVDs. Red fluorescence represents the migrated NPSCs into the IVDs. (c) Comparison of cell migration in each area of the IVDs among the three groups using the appearance rate. NPSCs: nucleus pulposus-derived stem cells; IVD: intervertebral disc; M: metaphysis; E: epiphysis; CEP: cartilage endplate; OAF: outer annulus fibrosus; IAF: inner annulus fibrosus; NP: nucleus pulposus. Values are presented as the mean ± SD (*n* = 5); ^∗^*P* < 0.05.

To track cell migration into the coccygeal IVDs in rats *in vivo*, PKH26-labelled NPSCs with or without pretreatment with AMD3100 were injected intravenously. After two weeks, the IVDs from the three groups were harvested for histological analysis. A large amount of the systemic injected PKH26-NPSCs were seen around the IVDs, but fewer cells appeared in the center of the IVDs under the fluorescence microscope (Figure 6(a)). The total number of the migrated cells in the degenerative IVDs of group B obviously increased, accompanied by the increased expression of SDF-1*α* (Figure 6(b)). The appearance rates in the OAF and NP areas increased significantly in group B compared to those in group A (*P* < 0.05), which was consistent with the distribution of the highly expressed SDF-1*α* in the degenerative IVD. However, there were no significant differences in the appearance rates between group A and group C (*P* > 0.05) (Figure 6(c)). This observation suggested that endogenous NPSC migration could be triggered by SDF-1*α in vivo* highly released from the degenerative or injured IVD.

### 3.5. Effect of SDF-1*α* on the Expression of CXCR4 in NPSCs

**Figure 7:**
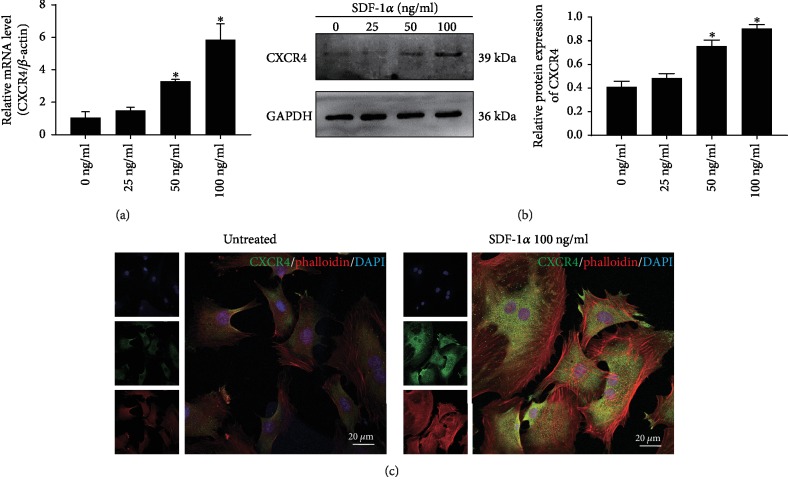
Effect of SDF-1*α* on the expression and distribution of CXCR4 in NPSCs. (a) Real-time RT-PCR analysis of the mRNA synthesis of CXCR4 in NPSCs. (b) Immunoblotting analysis of the protein expression level of CXCR4 in NPSCs. (c) Immunofluorescence analysis of CXCR4 (green) and F-actin (red) in NPSCs. SDF-1*α*: stromal cell-derived factor-1*α*; CXCR4: C-X-C chemokine receptor; NPSCs: nucleus pulposus-derived stem cells. Data are presented as the mean ± SD (*n* = 3); ^∗^*P* < 0.05.

Real-time RT-PCR and Western blot demonstrated that SDF-1*α* significantly increased the expression of its receptor CXCR4 in a dose-dependent manner (Figures 7(a) and 7(b)). Immunofluorescence analysis showed that CXCR4 was also expressed in the cytoplasm of NPSCs, which could be not only obviously upregulated but also transferred to the cell membrane by the stimulation of SDF-1*α*. In addition, we found that SDF-1*α* enhanced F-actin cytoskeletal organization and lamellipodia formation in connection with cell migration (Figure 7(c)).

## 4. Discussion

Stem cell therapy has been recognized as a promising strategy for IVD regeneration [27–29]. However, the long-term survival and biological function of the transplanted cells in the harsh microenvironment of the degenerative IVD remains controversial [30, 31]. To overcome the problems associated with stem cell transplantation such as the shortage of cell sources, the excessive cost, and the potential adverse events, mobilization and recruitment of endogenous progenitor/stem cells into the injured site have been considered alternative approaches [32]. Many studies have demonstrated that progenitor cells residing in the stem niches of the IVDs have the migration capacity toward the annular fibrosus and the inner parts that may contribute to IVD regeneration *in situ* [7, 8, 33]. Previously, our research team successfully isolated and identified NPSCs from rat coccygeal IVDs that have the capacities of self-renewal and multilineage differentiation [11]. The current study mainly demonstrated that the chemoattractant SDF-1*α* secreted in the degenerative IVD could stimulate the migration capacity of endogenous NPSCs using an *ex vivo* IVD organ culture model and a degeneration-induced IVD model *in vivo*.

In the natural healing process, the affected cells and tissues release a variety of cytokines/chemokines to activate the dormant progenitor/stem cells and then trigger the migration from their niches to the injured sites contributing to repairing the injured tissue *in situ* [34]. Some investigators revealed that IVDs cultured under degenerative conditions stimulated MSC migration towards the tissue [35, 36]. CCL5/RANTES was described as a key factor secreted from the IVD and involved in the recruitment of MSCs [35] and then was identified as an IVD degeneration marker in the plasma of patients with lumbar disc degeneration [37]. Similarly, the chemokine SDF-1*α* was upregulated in the process of IVD degeneration [19, 20]. However, few researches have been reported about the biological effects of the SDF-1/CXCR4 axis on the endogenous progenitor/stem cells within the IVD. The hyaluronan-based delivery of SDF-1*α* was proved to significantly boost the recruitment of MSCs into the degenerative IVD in organ culture *ex vivo* [22]. In another study, Zhang et al. [38] developed BSA/heparin nanoparticles (BHNPs) as injectable carriers for SDF-1*α* to slow down the degradation. They found that SDF-1*α*-loaded BHNPs could stimulate the recruitment of MSCs into the injection site for IVD repair *in vivo*. Therefore, it is necessary to determine whether the SDF-1/CXCR4 axis could attract IVD endogenous progenitor/stem cells into the degenerative sites contributing to tissue repair *in situ*.

It is believed that the released proinflammatory cytokines during degeneration result in the production of various chemokines. IL-1 and TNF-*α* recognized as the important factors for IVD degeneration [39–41] can be bound in the extracellular matrix surrounding the native cells within the degenerative IVD that causes their higher localized regional concentrations [42]. They participate in IVD degeneration by promoting extracellular matrix degradation, IVD cell phenotype changes, and chemokine production [43]. For example, some researchers reported that TNF-*α* and IL-1*β* synergistically induced CXCL16 production in endothelial cells, smooth muscle cells, and fibroblasts [44]. IL-1*β*-stimulated NPCs attracted and mediated the migration of MSCs [45]. A recent study has demonstrated that the stimulation of NPCs with IL-1*β* and TNF-*α* induces the production of a chemokine, macrophage inflammatory protein-1*α* (MIP) for macrophage migration in the degenerative IVD that would help attenuate further degeneration through phagocytosing the apoptotic cells [46]. The expression and release of CCL5/RANTES are correlated with the proinflammatory mediators IL-1 and TNF-*α*, which are known to be upregulated in the degenerative IVD [35, 39, 47, 48]. Moreover, the upregulation of the chemokine CCL3 was observed in NPCs after exposure to the proinflammatory mediators IL-1 and TNF-*α* [46]. These chemokines may promote both the recruitment of progenitor cells and infiltration of macrophages in degenerative discs [46]. Therefore, we investigated the presence of SDF-1*α* in the monolayer disc cell culture during exposure to IL-1*β* and TNF-*α*, mimicking the degenerative IVD microenvironment *in vivo.*

Our results demonstrated that the synthesis and secretion of SDF-1*α* were induced significantly when NPCs were cultured in the proinflammatory conditioned media using IL-1*β* and TNF-*α in vitro*. In addition, immunohistochemical staining showed the expression of SDF-1*α* was upregulated significantly in the degeneration-induced IVD model, which was consistent with the high expression of SDF-1*α* in human degenerative IVDs [19, 20]. These findings suggest that proinflammatory cytokines in the degenerative IVD may increase the production of the chemokine SDF-1*α* for the potential mobilization of the resident progenitor cell populations to facilitate IVD regeneration.

In addition to migration, SDF-1*α* is also important for the survival, proliferation, and differentiation [49]. For example, SDF-1*α* has been shown to promote the survival of oligodendrocyte and trophoblast precursors [50, 51]. Wei et al. [21] found that overexpression of CXCR4 could promote MSC retention in the degenerative IVD that enhanced the stem cell-based regeneration. In the present study, we also demonstrated that SDF-1*α* could improve the late proliferation of NPSCs. However, pretreatment with AMD3100, the CXCR4 antagonist, significantly inhibited the increased viability. These results suggest that the positive effect of SDF-1*α* on the biological viability of NPSCs may be mediated through the SDF-1/CXCR4 axis.

Although cumulative efforts have been made to delineate that SDF-1*α* is a good candidate chemokine for recruitment of MSCs, little is known about its effect on the migration capacity of NPSCs during the process of IVD degeneration. It was reported that MSCs could be efficiently recruited in an *ex vivo* organ culture model using a nucleotomized bovine IVD [22, 36] and the cavity allows hydrogels or solutions to be easily injected into the IVD [22]. Besides, the whole-organ culture model *ex vivo* has been verified to mimic the *in vivo* condition more closely [52]. In the present study, we also used the nucleotomized bovine caudal IVD to simulate the degenerative feature of the loss of NP tissue. To evaluate the migratory effect of SDF-1*α* on NPSCs, we analyzed the migration profile along with IVD depth from top to bottom. The random migration in the subsections closer to the upper CEPs was observed in the control group, suggesting that the migration might be triggered by gravity or the cavity in the NP tissue. In the middle or even deeper subsections, the number of migrated cells was evidently increased in response to SDF-1*α* in a dose-dependent manner. These results suggest that NPSCs have the migration capacity though the CEPs and extracellular matrix, although to a limited number, the presence of SDF-1*α* in the *ex vivo* organ culture model of the nucleotomized IVD can enhance the migratory capacity of NPSCs for tissue regeneration.

To support the concept that the endogenous NPSC migration is regulated by SDF-1*α* that secreted by the native cells in the degenerative IVD for regeneration *in situ*, we transplanted NPSCs labelled with PKH26 intravenously, with or without pretreatment with AMD3100, into the rats that had underwent the surgical procedure for coccygeal IVD degeneration, and then compared the magnitude of differences in the distribution of the labelled cells between the normal and the degenerative IVDs. Using this model, we traced the migrated NPSCs into the IVDs in response to the increased SDF-1*α* during the progression of IVD degeneration *in vivo*. Considering the blood stream direction from the vertebrae toward the IVD, exogenous NPSCs must firstly cross the growth plate, then through the epiphysis, and lastly into the canals of the endplates. The results of fluorescence showed that NPSCs could be found in all these regions and the cell distribution was significantly different between the control group and the degeneration group. In the control group, the magnitude of NPSC migration gradually decreased with increased distances. The marked cell appearance rates in the OAF and NP areas could be found in the degeneration IVD, which was consistent with the distribution pattern of the upregulated SDF-1*α* observed in the degenerative IVD. After pretreatment of AMD3100, the magnitude of NPSC migration was decreased and the distribution was not different from the control group. These findings suggest that the degenerative IVD releases the chemokine SDF-1*α* that is effective on the recruitment of endogenous stem cells *in vivo*.

The binding of SDF-1*α* to its receptor CXCR4 regulates chemotaxis, cell survival, and/or proliferation by stimulating numbers of downstream signal pathways. The collected evidence demonstrated that MSCs expressing CXCR4 have the powerful chemotaxis to SDF-1*α* and SDF-1*α* can promote the migration of the BMSCs by increasing the expression of CXCR4 [53]. The present study demonstrated that the expression of CXCR4 was upregulated by SDF-1*α* in a dose-dependent manner but effectively inhibited by AMD3100. The majority of CXCR4 is localized in the cytoplasmic region of MSCs, and it cycles continuously to the cell surface and reenters into the cytoplasm via endocytosis [54]. The immunofluorescence revealed that CXCR4 was mainly expressed in the cytoplasm of NPSCs, and SDF-1*α* not only increased the CXCR4 expression level but also induced CXCR4 to transfer to the cell surface. This phenomenon was also appeared in the regulation of the SDF-1/CXCR4 axis on the chemoattraction to MSCs, which suggests that SDF-1*α* impacts cellular CXCR4 production and its surface expression [55]. Furthermore, we found that SDF-1*α* promoted the formation of F-actin and lamellipodia in NPSCs that suppressed by AMD3100. F-Actin cytoskeletal networks have been widely accepted as key regulators of cellular shape and force generation in cell migration, which are involved in the lamellipodia formation, cell adhesion, and cellular shape changes [56]. These results suggest that SDF-1*α* induces the production and distribution of CXCR4 and the rearrangement of F-actin cytoskeletal networks in NPSCs, leading to enhance their migratory capability.

There are limitations in the present study. Firstly, we assessed the biological effects of SDF-1*α* on the *in vitro* expanded rat NPSCs. However, the cultured cells *in vitro* scarcely behave in the same manner as *in vivo*. Secondly, some certain limitations should be taken into consideration when comparing the condition in the nucleotomized IVD model to that in the human degenerative IVD, although this *ex vivo* model described in the present study has been validated in a previous work [36, 57]. Thirdly, in the animal experiment, we transplanted the exogenous PKH26-labelled NPSCs into the rat intravenously for the convenience of cell tracking. Labelling the endogenous stem cells in the IVD with specific markers *in vivo* is needed to be performed to support our hypothesis that NPSC migration could be enhanced by the upregulated SDF-1*α* in the degenerative condition involved in the process of IVD regeneration *in situ*. Although our study showed the important role of the SDF-1/CXCR4 axis in NPSC migration, a future work will focus on the possible downstream signal pathways of the SDF-1/CXCR4 axis that regulate NPSC migration while elucidating the contribution of NPSC recruitment to IVD regeneration.

## 5. Conclusions

In summary, our findings indicate that the chemokine SDF-1*α* is produced by the intervertebral disc cells, which is increased in response to the degenerative condition. The SDF-1/CXCR4 axis effectively promotes the migration of the endogenous stem cells residing within the IVD, which provides novel strategies for self-repairing in the early stage of IVD degeneration.

## Data Availability

The data used to support the findings of this study are available from the corresponding author upon request.
